# The Hospital Nursing Supplement

**Published:** 1894-06-16

**Authors:** 


"The Hospital" Nursing Mirror.

Being the Extra Nursing Supplement of "The Hospital" Newspaper.

[Contributions for this Supplement should be addressed to the Editor, The Hospital, 428, Strand, London, W.C., and should have the word "Nursing" plainly written in left-hand top corner of the envelope.]

### News from the Nursing World.

### PRACTICAL CHARITY.

The Invalid Children's Aid Association requires more visitors, and it ought to get them directly the want is made public. Whatever differences of opinion may exist with regard to other associations, everybody must agree as to the claims of sick children. The Invalid Children's Aid Association looks after little invalids of all kinds, endeavouring to lighten the bodily suffering and mental weariness of the frail little creatures, whose sickliness is aggravated by poverty. The association has its headquarters at 18, Buckingham Street, and application can be made there by anyone to have a crippled or invalid child looked after. A visitor is found for each case, whose business it is to find out first what is wanted, and then to supply it. Spinal couches, perambulators, letters for convalescent homes or hospital, warm clothes, nourishing food, toys, sheets, and even cots are forthcoming. Systematic visiting is of the greatest help to the children, and therefore ladies are always in request to undertake it. If they have some practical knowledge of district nursing, so much the better for the children and for the mothers, who are often willing to be taught how to mitigate the sufferings of the "afflicted" little one. At any rate, a kind, intelligent, persevering visitor is always in demand, and not the sentimental young lady who only likes "pretty children, you know, in nice, clean cottages." The homes of the London poor are very often neither pretty nor clean, but as small invalids have to live in them, surely their luckier sisters can spare time and patience to visit them.

### DISSATISFIED.

The nursing arrangements at Hampstead Workhouse do not appear to go very smoothly as yet. Lately a memorial was sent to the Board, according to the local press, by the nurses. They consider their salaries too low, and therefore they demand "table money," which is apparently the new term for the "beer money" of the past. A complaint that they have to take double duty was found to relate to the extra work incurred when any nurses had leave of absence. The application for beer money was not acceded to, but we should be glad to have a clear explanation as to the amount of so-called extra duty which each nurse is liable to. This is a matter which concerns the comfort of the patients as well as the health of the nurses, and therefore it is of interest to all concerned that the statement which was introduced in the memorial should be carefully considered by the board and their medical officer with a view to elucidating the exact hours on duty of each member of the nursing staff, and so proving whether there is anything unreasonable in the existing arrangement.

### NURSES AT PETERBOROUGH.

The Peterborough District Nursing Association appears to do its work with commendable thoroughness. The nurses' duties are carried on under the doctors by whom they are summoned to cases requiring skilled attention. The association is affiliated with the Queen's Jubilee Institute, and the nurses' responsibilities include not only the special charge and personal cleanliness of each patient, the care and ventilation of the sick room, but the instruction of the relatives of the sick how to keep the room in nursing order, and to encourage self-dependence. At the annual meeting in May, the clergy and doctors were specially cordial in their acknowledgments of the excellent work of the superintendent and nurses, Dr. Cane saying "neither the town nor the doctors could possibly do without them now."

### LONG NIGHTS.

The entire absence of night nursing at the workhouse has been pointed out in the course of the inquiry at Newton Abbott. The sick have apparently passed the weary hours without any competent attendance. It has been suggested that not only ought a second day nurse to be engaged but a night nurse also. One guardian is stated by the local press to have objected to this, saying, "Too many officials in a house is not a good thing; they lead to so much bickering. The third nurse ought certainly to sleep out of the house if appointed." This view of the discipline of trained nurses is somewhat unflattering and unusual. With regard to the "sleeping out," it is acknowledged that a proper measure of rest and sleep is seldom secured by any nurse who is thus circumstanced. To look back on the long years when the unhappy paupers, whether sick, feeble, imbeciles, lunatics, or little children have been left altogether during the night watches uncared for in darkness, is such an appalling retrospect that instinctively most of us shun the mental ordeal and prefer looking forward to the installation of increased and competent nursing staffs in every Poor Law infirmary.

### PROPOSED INCREASE OF NURSING STAFF.

A considerable addition to the Nurses' Home at the Brownlow Hill Infirmary is under contemplation. The Local Government Board Inspector, in reporting very favourably of the condition of the institution, advised an increase of the staff, especially in the matter of night nurses. The Liverpool authorities, having gone thoroughly into the question, have wisely decided to have twenty additional nurses, and to build adequate quarters for them. It is easy to believe that the suggestion made by the Nurses' Committee will be carried out in their entirety—that a new building, "complete in itself and up to date," will be the eventual result.

### AN ALARMING STORM.

An unprecedented hail-storm occurred at Vienna last week, which left the city looking "as if a hundred explosions had taken place at the same time," not only enormous injury to property, but considerable injury being done to peopl·, as well as animals. Many birds were killed, the terror displayed by the horses being pitiable. At the Great Central Hospital it is said that ten thousand panes of glass have been shattered, and the difficulty of keeping up the temperature of the wards has been very great, although efforts were immediately made to convert blankets into effectual temporary screens. During the progress of the storm the patients on the exposed side of the hospital were terribly frightened by the hailstones, which penetrated through the damaged window sashes. Those who could walk were promptly assisted to reach more sheltered spots, and the beds of the helpless patients were carried bodily into the corridors.

### THE REGISTERED NURSES' SOCIETY.

We are assured, by an esteemed correspondent, that no nurses will be admitted to this society, although they may be "registered," unless they have spent three years' in a hospital of forty beds or upwards.

### A PLEASANT AFTERNOON.

Such an attractive programme was provided, that those present in the Queen's Hall on the 7th inst. at "Mrs Brewer's Afternoon" will not easily forget it. The North-Eastern Hospital for Children, Mrs. Starey's "Blind in their Homes," the Cripples' Branch of the Ragged School Union, and the Poor of Spitalfields are the charities whose funds benefit by this entertainment. Madame Antoinette Sterling gave immense pleasure by her songs, and she kindly acceded to the vigorous encores with which each was greeted. Madame Alice Gomez's charming performance was received with equal cordiality, and Mrs. Alfred Barker's clever recitations won great admiration. The unique feature in the entertainment was provided by Miss Gethen (late Sister Queen). This consisted in the relation of an incident of ward life occurring in the narrator's own experience. The story in itself was full of a deep pathos and significance, and the dramatic manner in which Sister Queen told it was above criticism. Madame Sarah Grand was welcomed enthusiastically when she came on to read the proem to "The Heavenly Twins," and the fine playing of M. Holeman and Signor Caprile were appreciatively listened to by a most interested audience. The songs of the Scandinavian Quartette and the Schartau Part-Singers were equally enjoyable.

### MELBOURNE.

A long discussion has taken place at one of the Melbourne hospitals on the subject of kerosene, one member of the managing committee asserting that a saving of £100 per annum would be accomplished by the substitution of lamps for gas. It was urged, however, by other gentlemen that the introduction of kerosene would be fraught with danger to the structure, the floors being polished and highly inflammable. The president was in favour of the introduction of electric lighting, and it is probable that his proposal will receive the attention which its wisdom deserves.

### NURSES AT PHILADELPHIA.

Designs for a handsome new nurses' home were exhibited in the amphitheatre at the "Commencement" of the Philadelphia Hospital Training School held last month. Seventy nurses in uniform, and nineteen graduates, were the centres of interest on the occasion. Philadelphia Hospital has been in existence for over 150 years, and the excellent Nursing School, founded in 1885, will always be associated with the names of Miss Alice Fisher and Miss Horner. As one of the speakers at the Commencement remarked, "Nor is its reputation limited to this country, for in England, at least, this school is frequently the subject of favourable comment." The hospital always contains over a thousand patients.

### "A DREADFUL ERUPTION."

The foolish habit indulged in by certain private nurses of discarding uniform when with a case does not appear to be restricted only to Englishwomen. Dr. Weir Mitchell, whose warm interest in the progress of nursing is well known, remarked in the course of an address at Philadelphia the other day, "I note with annoyance how few nurses keep to their cap and apron. I have begun to associate a good conscience with a clean apron. When I have to find fault, it is very apt to be with some young woman who has given up her pretty and becoming uniform, and broken out with her first money into a dreadful eruption of gowns, after the last silly and ugly fashion. Pretty soon the fine gowns and frills and bits of lace at the wrists get into the room where the patient is, and surely more unfitting things could not be. Let me beg of you to dress simply and neatly, and to put by all you can for the stormy days, which come to all lives soon or late."

### MUTUAL BENEFIT.

The Mutual Benefit Association of Nurses in New York is collecting a fund to provide sick pay for members of the Nurses' Club. It is proposed to allow five dollars a week for twelve weeks in the year as required.

### GRADUATES AT BROOKLYN, U.S.A.

On the occasion of the awarding of certificates to the graduates of the Brooklyn Memorial Training School, an excellent report was given by Mrs. Georgia Cassidy, M.D., the secretary. She took the opportunity of congratulating the nurses on the presence of mind they had displayed at a recent fire, when thirty patients had been removed from the burning building to the neighbouring training school without damage or alarm to any one of them. The president, Mrs. J. H. Burtis, gave an interesting address before handing their certificates to the class. She said "Don't be merely professional. Do not look upon patients as made for nurses, but rather remember that nurses are made for patients. . . . Keep yourselves in sympathy one with another and with those who have preceded you. . . ." An excellent programe of music was carried out, and greatly appreciated by the large party of guests who had assembled in honour of the occasion.

### USEFUL DEVELOPMENT.

For some time past a Nursing Home has been connected with the Wotton-under-Edge and District Nursing Association. The report at the last annual meeting showed that such development was taking place that it was decided to take a larger house in the summer, and convert the Home into a Cottage Hospital and Convalescent Home. The new institution will prove all the more popular from the fact that the nurses have become well-known and their services appreciated in the neighbourhood.

### SHORT ITEMS.

A successful conversazione and concert were recently given at Northampton, in aid of the funds of the District Nursing Association, which is affiliated with the Queen's Jubilee Institute.—The Holborn Guardians have decided to have a qualified receiving ward nurse in place of a porteress.—An excellent concert was given by the students and their friends at the London Hospital Medical College, on June 12th.

### On General Nursing.

By Rowland Humphreys, M.R.C.S., L.R.C.P.Lond.

### XVI.—RHEUMATIC GOUT.

The disease which goes by this name is, oddly enough, neither rheumatism nor gout, although it may be complicated by either or both of these complaints. It is a disease in which the nervous system plays a large part. By rheumatic gout, in this article, is meant a disease of the joints, whose onset and course is associated with, or even marked by, a number of nerve complications. It is a progressive malady which may attack people at any age, terminating in complete disorganization of the joints attacked.

In the early years of this century the disease, which had long gone by the name which stands at the head of the article, was shown to be a disease separate and distinct from any other, and although medical men of those times did not know how to meet it, nor how to treat it with any success, yet to separate it from other diseases was a great stride. The patient in a bad case of long standing is usually confined to bed, the joints are fixed, distorted, enlarged, more or less tender. Round the sockets may be felt lips or edges of bone, which have grown up in the course of the disease. At times these joints are attacked with inflammation, and ache so that the patient is kept awake at night, perhaps night after night. The distortion and "erosion," or flattening out of the joints, is so great that the fingers are often dislocated at the knuckles nearest to the palm, and the ends of the bones project beneath the skin. In many cases the joints become rigid, the capsule or sheath of the joint becoming calcified or hardened, and thickened, the surfaces of the joints adhering together.

In an earlier condition many of the joints, especially those of the hands, are enlarged in a turnip-shaped manner by the fluid which collects in them, and with the effusion into the tissues round them makes up the typical fingers seen in these cases. The skin is more or less red, and from one attack to the other there is no real intermission, the tenderness and swelling more or less continuing.

During the acute attacks the temperature rises, and there are other signs of constitutional disturbance; but, unlike rheumatic fever, there is no particular sweating, and the tendency to attack the heart seems to be absent. The more chronic the case the less the fever, but there is less tendency for the joints to improve between these attacks. Another point in which these cases differ from acute rheumatism is that the inflammation does not wander from joint to joint.

There is a very considerable degree of anæmia in this complaint, and the patient is subject to local or general sweating in a manner very similar to that seen in phthisis. The heart is usually rapid and the pulse hard.

At an earlier stage than this the patient complains of occasional tenderness in and inflammation of one or more joints, of general weakness, of a constant feeling of being tired, with loss of appetite, emaciation, and constipation. Wasting of muscles and ligaments is very common, and seems often to precede the joint disease. The outer or back surfaces of the forearms and similar parts of the skin are marked in a curious way with freckles or even with large patches of pigment, especially on the neck, so that it always looks dirty.

Garrod divides the disease into three forms, the acute, the chronic, and the irregular, the former being very rare, and of the nature of one acute attack in which the joints become more or less irremediably injured; while the chronic form is that in which the changes in the joints are spread over a long period of time, and with many attacks of inflammation. If the patient be not under favourable surroundings, the disease tends to spread, and, attacking one after another, implicates nearly every joint in the body. The jaw seems to come in for injury in almost every case, and, as this hardly ever happens in gout, it has been made a point of diagnostic difference between the two complaints.

Other characteristics of the disease are the little nodes or hard swellings seen especially on the fingers near their terminal joints. These are caused by the outgrowth of bone characteristic of the complaint.

Depressing causes associated with insufficient food (either because the supply of food was insufficient or because the nourishment was not assimilated) appear to be the chief factors in the causation of the complaint.

Sometimes the pain in the joint is relieved if it be buried in very hot sand for an hour or two at a time two or three times a day, or the joint affected may be slung or the limb fixed on a splint. The diet should be carefully attended to, avoiding excess and abjuring malt liquors and indigestible dishes.

Flannel must be worn, and the ffected joints wrapped in cotton wool or flannel. Sometimes turpentine liniment relieves.

Patients with this disease suffer from vague though sufficiently severe pains in the stomach, and, as there is often loss of sensation in the feet, it is very like locomotor ataxia in many, but, unlike the latter disease, the reflexes are not lost. In locomotor ataxia the joints at times become disorganised, and, as in rheumatic gout, loose bodies composed of fibro-cartilage are often found in the joints.

Rheumatic arthritis, another name for this complaint, proceeds in many cases from, or is subsequent to, an attack of rheumatism, though occasionally it appears to be an entirely independent disease Some authorities consider it a rheumatic complaint pure and simple; others deny this, and others attribute it to the development of a phthisical strain. At any rate, whatever be the cause the result seems to be much the same, and the treatment is similar. When the complaint has once become well marked nothing seems to arrest it. In order to do real good it must be attacked at a very early stage, and even then it is a hard matter to deal with successfully.

The first thing to be done towards a cure is to try to get the patient into a good condition of health. For the anæmia iron with arsenic or strychnia have to be given. Rest is essential, and the joint which is first affected is that which is habitually most used. Hence the habits have to be more or less changed. Galvanism, massage, a course of treatment at Bath or Aix, or perhaps a sea voyage may do much good.

Cod-liver oil is invaluable, especially if combined with iodides. Painting with iodine or blistering will often help the recovery of a joint. For pain various applications may be used, or the joint may be strapped. The treatment in the early stage is directed towards getting rid of the complaint —in the later ones towards obtaining relief. Nothing relieves like a stiff joint, and though the patient should, in the early stages, move the joints regularly and systematically, yet in the late ones it appears better to try to get a fixed joint.

During the acute attacks, quinine or salicylate of soda, and perhaps such drugs as antefebrin and antipyrin will relieve. Hot applications to the inflamed parts, or glycerine of belladonna be smeared over the joint, or belladonna liniment, with morphia dissolved in it, may be used to relieve the pain. Spirit lotion on lint covered, in this instance, with oil silk, or blistering may also be employed. In the chronic form a vapour bath or douche applied to the affected joint may be used. Rubbing the joints with goose grease, or some similar substance, is also very helpful. In very chronic cases the more the joint is fixed the easier will life be for the patient, and, after much disorganisation has taken place, it is useless to worry the patient with attempts to move it; the best that can be hoped for is absolute fixation. The application of plasters help this somewhat.

### The Mechanism of Movement.

### III.—STRUCTURE OF BONE.

Endless other illustrations might be brought forward, but enough has been already said to prove that bone is a most admirable material from the engineer's point of view for tubes, that a tube is the strongest possible form, and the one most frequently chosen by an engineer; that to obtain the greatest amount of strength combined with lightness—an all important matter since we naturally desire to move about a good deal—a hollow tube is preferable to a solid bar, because strength depends on arrangement of material, and not on its actual amount.

We have also seen how carefully the stronger material, the "compact" bone is arranged on the outside of the bone, where the greatest strain is found, and that it is increased in amount, though to a small extent, at those points where the strain is greatest, the bone being of the cylindrical shape. So much for the "shaft" of the femur; but now as regards this crane-like part at one end, technically called the "head." Is there any reason why it should be this peculiar shape?

This is the only bone in the body which has to support the same amount of weight and in this special manner. The other long bones do not. The lower part of the leg below the knee consists of two distinct bones (tibia and fibula), which share the weight. In the arm there is no special weight to be supported, because the strain is that of a pulling force, or if a heavy weight be held in the hand, of a sustaining force; but there is no weight at the shoulder at all comparable to that at the hip. Hence, whilst the humerus has a more or less rounded "head" to meet its special use, the femur has a "head" and a "neck," the protuberances between the neck and the shaft being technically called the great and small "trochanters," and the whole forms an end somewhat resembling a crane.

Now this arrangement has its exact counterpart in what the engineer calls a "flange." That is, when a beam of iron is made to support a weight, it is shaped like a flange, the extra strength is got just where required, viz., near the weight to be supported. Hence a beam is obtained of the right strength and there is no waste of materials, where strength is not needed. Had the beam been made all the way down as wide as the top or "flange," it would have been very clumsy, very heavy, and the extra material would be wasted. Exactly the same principle is carried out in the femur. The "head" corresponds with the "flange" in the beam, it receives the weight of the trunk, and this is distributed throughout the shaft. This arrangement, combined with the natural strength of bone for bearing equally well both tension and pressure, enables us to withstand the variety of strains in every possible direction, to which our limbs are subject, and they are neither very liable to break, nor are they heavy and unwieldy.

The "short" differ from the "long" bones in this respect, that they are not tube-like, but resemble pillars rather, the cancellous or lace-like bone occupying the whole of the interior; the same holds with regards to the ribs. As a complete contrast to the femur, take the scapula; this is a very light bone, as would be expected from its position and use. It would be extremely inconvenient to us to have a heavy weight pressing us down where now our light shoulder-blades are placed. The scapula has very little weight to bear, and is capable of comparatively little movement. Hence a heavy, strong bone, similar in substance to the femur, would be completely out of place and worse than useless.

The object of both the scapula and of the pelvic bones is to support and protect the delicate and vital organs contained within; the scapulæ protect the lungs at the back, and the pelvis the organs contained in the abdomen.

Thus these bones are composed of a thin outer layer of compact tissue, with the cancellous tissue between, like a sandwi h, and they are comparatively thin, and are hence called "flat." Another "flat" bone, but of totally different type, is that of the skull, where we find the strongest bones in the body, since their function is to protect one of the most important of all the organs—the brain. The skull, moreover, does not consist of one bone only, but of no less than eight different ones, excluding those which form the face. These are most firmly welded together by means of what are technically called "sutures," viz., projections like the teeth of a saw indenting the edges of each bone, and fitting in so securely that it is an extremely difficult matter to separate them, requiring considerable force.

The skull bones are composed entirely of compact tissue, and hence their great strength. Each bone is slightly convex, giving the whole when united the rounded shape of the skull, and also its great resisting force, affording the most effective protection to the brain within.

The weight which can be borne by the head is proved by the heavy loads which can be carried on it without the slightest harm, and this alone proves what a very strong protection has been given to the organ on which depends our power of movement, besides all our special senses, so that injury to the brain, or any part of it, means injury to some very important function.

The forms of the skull distinguish different races of mankind, and are usually divided into three groups: (1) long-headed, with more length than breadth, as the negroes and native Australians; (2) short-headed, which are very short compared with their breadth, viz., the North American Indians; and (3) medium-headed, where neither breadth nor length is extreme, as in the Central European, the French possessing the typical form.

Mere form alone is no guide to intellect, or the French nation would bear the palm. What is important is the capacity of the skull, and even this gives no accurate estimate, since the quality of the brain itself is the only true guide.

Man alone of all vertebrate animals bears his head erect. Other animals have projecting heads, and the jaw bones especially much longer, to enable them to get their food, because their fore-limbs have to help them in locomotion, while man's arms are free to convey food to the jaws, and he can always retain his upright position.

In early life the bones of the skull are soft and compressible, and therefore its shape can be easily altered, as is done by some barbarians, certain Indian tribes of North America, who are not content with the shape of head that nature has given them, and who place their infants in tubs with a hard, heavy thing on the head fastened down severely to the sides of the tub, and the infant's head is flattened and lengthened from back to front. We are quite as cruel as those Indians when we put heavy bonnets on infants' heads.

The reason why an infant's head is easily compressed is that it is formed of eight bones not welded together into one mass, the sutures loosely fitted in, and between some of the bones is a space merely covered by a tough membrane, where the throbbing of the blood in the brain below is seen. This arrangement is nature's provision for the increase of the brain within with the child's growth. Were the baby born with a full-sized head, it would be a very heavy load for its poor little weak body to support. Hence the need for the utmost care when handling an infant's head with avoidance of blows or pressure, and any attempt to press together these bones which nature has left apart under the impression that it is good for the infant

### The Royal British Nurses' Association.

There has been some difficulty experiencd in procuring copies of the *Nurses' Journal*, the official journal of this association, for May, which is now, we understand, removed. The *Nurses' Journal* is a reputable, outspoken, and interesting quarterly, which we hope will continue to thrive and prosper. The May number contains a leading article which all nurses and all who are interested in their welfare and progress should read. It is entitled "The Work and Wants of the Association," and is full of sober sense and well-reasoned truths. It points out that the Royal British Nurses' Association "has now reached a period in its history at which it has become necessary to separate the chief ends of its existence from those of a secondary description, and to estimate the resources which are available for the fulfilment of either. The chief aims are, first, to insist that a hospital training of three years' duration is absolutely necessary as a preliminary to assuming the title of nurse; and, secondly, to unite all women who have obtained this training, and whose conduct has been satisfactory throughout its course, into a society for mutual help and advantage, and for the better accomplishment of the work of their calling." The article proceeds to point out that to attain these objects it is essential that the association should possess "influential support, public recognition, a reasonable and moderate command of funds, and a sufficiently sagacious internal administration."

Influential Support and Public Recognition.

The presidency of H.R.H. the Princess Christian, it is justly stated, "supplies everything which is attainable" in regard to the two first essentials, providing the patronage thus graciously extended is cordially met by the loyal fulfilment of "the obligations entailed upon them by whom it is received. A royal personage who condescends to identify herself closely with any institution is regarded by the public as being to a great extent responsible for its management; and hence it is essential that in this management, even if only as a matter of simple justice, the wishes of such a person should prevail. A princess would be unable, as a general rule, to continue in the presidency of an association in which her opinions were over-ruled by those of others, or in which the conduct of business was disturbed by questions arising out of personal susceptibilities. . . . Until this condition (*i.e.*, a clear perception of this fact) is fulfilled, the next one we have mentioned, that of public recognition of the usefulness of the institution concerned, cannot be looked upon as secure."

These contentions are sound as a mere matter of abstract principle, but when they are applied to the Royal British Nurses' Association they have ten times additional force, because H.R.H. the Princess Christian is, and always has been, the life and soul of this Association. But for Her Royal Highness it would never have been established, it could never have obtained its Royal charter, nor but for her would the eminent members of the medical profession whose names appear upon the title page of its report have lent to it the weight of their position and support. H.R.H. the Princess Christian has ever been the one person upon whom the chief responsibility and work has rested, and without her, as we have said, the Royal British Nurses' Association never could have existed. Nor is this all, for Her Royal Highness has thrown herself in the most self-denying way into the work, and however much many may have differed from her views, even her enemies (if she have any, which we very much doubt) must be full of admiration for the zeal, devotion, and pluck which she has displayed throughout a long and most arduous controversy. We cannot imagine that any members of the Royal British Nurses' Association could ever display sufficient ingratitude or temerity to set themselves in opposition to H.R.H. the President, to whom they owe everything, but should they ever do so we have enough knowledge of the sterling character of British nurses to know they would unite as one woman to promptly turn such traitors out of their camp, be they who they may.

A Reasonable and Moderate Command of Funds.

On this head the writer in the *Nurses' Journal* declares: "The retiring treasurer leaves no debts to his successor, but he leaves an empty treasury; while at the same time the association cannot continue its work except at considerable increase of expense. The new rooms involve a higher rental, and the lectures and other arrangements, although likely ultimately to be remunerative, have in the first instance to be paid for. A proposal is now under the consideration of the executive committee by which, if it be agreed to, it is believed that the money required for these and other purposes, and generally for the extension and improvement of the work of the association, *may be made a charge upon its future revenues.*"

A Sufficiently Sagacious Internal Administration.

The article concludes thus: "Nurses will never obtain their deserts as long as they permit themselves to be divided; but it is an essential preliminary to the union which is desired, that the government of the association should be committed, at this period of its history, to persons who have no record of past strife, however brilliant or successful; who may easily be regarded as impartial, desiring to work with and for the benefit of all concerned, and whose sole desire will be not to triumph over an old adversary, or to secure another victory in a controversy worthy only of being forgotten, but to unite competent and properly-trained nurses, wherever they may be found, or wherever their training may have been received, in the bonds of a perfect and harmonious co-operation. The attainment of this great end should now be the chief aim of the association, and will, we trust, before long constitute its chief claim to the favourable consideration of the public."

We welcome this article because of its outspokenness and sterling sense. It breathes the spirit of goodwill and earnest purpose, and sounds a welcome note which is likely to meet a responsive echo throughout the world of nursing. *Neither parties nor persons, but the greatest good of the greatest number of nurses throughout the British Empire.* That is a sentiment which will carry H.R H. the Princess Christian to victory, because all who have the true interest of nurses at heart can unite cordially under such a banner, and all other persons do but constitute the tares of the nursing field, and may so be thrust out without hesitation or delay.

### Appointments.

[It is requested that successful candidates will send a copy of their applications and testimonials, with date of election, to The Editor, The Lodge, Porchester Square, W ]

York County Hospital.—Miss Anna A. Gwyn has been appointed Matron of the York County Hospital. She was trained at Winchester County Hospital, worked at Netley and St. Bartholomew's, was Matron at the General Hospital, Birmingham, for nine months, and held a similar post for five and a half years at Worcester General Infirmary. For the last three years Miss Gwyn has been Matron of the Harrogate Bath Hospital, and many good wishes accompany her to the new work for which her previous large experience has specially fitted her

### Poplar Hospital for Accidents.

Anticipation.

Regardless of the sudden showers which characterised the weather on Sunday, June 10th, hundreds of men, women, and children sauntered along the pavements and thronged the roadway which leads from Aldgate to the East India Docks. There was not much to be seen beyond flags and a little gay drapery festooned on some of the house fronts, but there was the knowledge of gay doings to take place on the morrow, and the prospect had visibly raised the spirits of the East-enders, causing a welcome break in their monotonous lives.

"'Ow many of 'em's a-comin'?" asked a woman whose large family of small children clung round her tattered skirts whilst she elbowed her way through the good-tempered crowd.

"Oh ever so many," was the rejoinder. "Why the Duke o' York's comin' with the Prince o' Wales. I'm a goin' to start my washin' at four to-morrer mornin' so as to git through an' 'ave a reg'lar day o' it. Why the Princess 'ud be a reg'lar treat by 'erself, let alone the young ones and the Lord Mayor."

Outside the Hospital.

Just outside the dock gates, at a junction of two roads, the hospital, with a notable air of newness about its exterior, exhibited a fine flagstaff ready to hoist a welcoming banner.

The inevitable awning and temporarily covered-in side entrance were the other outward signs of festivity.

Ascending the steps to the front entrance a friendly spectator warned us: "You're too late. The visitors isn't goin' in now. If it's a new case you wants, o' course you're all right." Then, after a pause, "I should advise you to go in at the accident entrance. You'll find a porter there in a minute." And so the good advice was followed.

The road slopes down to this doorway, obviating the necessity for steps. Patients are taken straight in to a spacious room, where every convenience awaits them for transit to "lift," and thence to the ward.

The enthusiasm of everybody connected with the little hospital forms a most conspicuous characteristic. From the president and chairman it extends to the porters and the wardsmaids, one of whom was heard to remark over night, "I'll come extra early on Monday morning to make sure as I've cleared everything away ready for 'em."

In the wards, however, on Sunday, there was no sign of special preparation. Why should there be? At any hour of any day in the week, not only the wards but corridors, kitchens, and other offices are perfectly fit for inspection by Royal or other visitors in a well-managed institution.

Of the structure of the hospital we have already given a description in our columns, and also of the excellent bedsteads with movable backs, and the lockers and dressing tables, each bearing the stamp of up-to-date inventions. In each ward stands a newly-added sister's table, where necessary writing is done in the centre of the work to which it relates. "What a pretty table," is the involuntary comment. "Yes, is it not? That is Mr. Holland's last gift to us." "He seems to make rather a pet of this hospital," we venture to remark, suggestively. "He leaves nothing out," is the fervent reply. "He cares for everything and everybody."

Women and Children.

Now at last women and children can be treated in the hospital, which during its forty years of existence has only been able to accommodate men, and one of the fine new wards containing ten beds and ten cots had already occupants.

A Welcome Gift.

In another ward a beautiful little cottage piano stood open, and witnessed to the genorsity and kindliness of the friend who had presented it. The small, separate bed-rooms of the nursing staff looked very pretty and home-like, and the nurses' sitting-room is a delightful apartment. The accommodation for the residents will make them envied by many other hospital staffs, for it is as good as possible, and in almost startling contrast to the wretched rooms which in many institutions are allotted to the medical officers.

Service in the Wards.

Lingering outside a ward door till the service was over, we heard the last verse of a hymn sounding infinitely touching and sweet. These "accident patients" are cheery fellows as a rule, and tell you of their mishaps and their "excellent recoveries" in a most interesting and simple fashion. The "good tone" of the place is also particularly evident. Everyone seems loyal, kindly, and courteous.

Monday at Poplar.

The fine marquee it was full to overflowing, and there were rows of sisters and nurses in their pretty uniform.

The Arrival.

The Prince and Princess of Wales, who were accompanied by the Duke of York and the Princesses Victoria and Maud, were received at the entrance and conducted to the platform, which was charmingly decorated with white marguerites, pelargoniums, and maidenhair fern. A lovely flower bouquet of pink roses tied with long streamers of pale green ribbon was presented to H.R.H. the Princess of Wales, by the little daughter of Mr. Hills, the president. Princess Victoria and Princess Maud wore fawn-coloured dresses and hats to match, and the dress of the Princess of Wales was black, with a small cape relieved with white lace insertion.

The Archbishop of Canterbury offered up prayer, which was followed by two verses of a hymn, and then Mr. Hills read an address in which he pointed out the great interest taken in Poplar Hospital by working men, one-sixth of the whole subscriptions being raised by them, chiefly in pence, proving that thousands of them were interested in the what they called "their own hospital." £30,000 had been collected in three years, and £4,000 of which was directly due to the influence of the Royal visit. The hospital was entirely free from debt. Mr. Hills considered the prosperous condition of the hospital was greatly owing to the energy and devotion of the chairman, the Hon. Sydney Holland.

The Prince of Wales, in reply, said: It has given the Princess of Wales and myself great pleasure to come here to-day, and I thank you all on her behalf for the kind reception you have given us. It will, I am sure, be readily understood that I am able to accept but a small proportion of the numerous invitations which I receive to be present at the opening ceremonies of institutions; but my deep and especial sympathy is with work of the nature of the Poplar Hospital, and when I was asked to open it I was glad to find it was in my power to return an affirmative reply. I have had the satisfaction of serving on the Royal Commission on the Housing of the Working Classes of this great Metropolis, and have been deeply impressed by the evidence of the miserable surroundings of the homes of so many of my countrymen. It may take some years before any really adequate measures can be adopted to lessen this evil, but in the meanwhile it is most encouraging to see that hospital accommodation is increasing in London, although it is far below the wants of the poor. It will, indeed, be a happy day when every poor person in London who cannot afford attendance at home has the best prospect offered him of a quick recovery from illness in a thoroughly well-administered hospital. I can imagine no charity which should appeal more strongly to the rich than this hospital for it possesses, among other advantages, that of being situated in the very district where it is most needed—among crowds of poor and hard-working men. I can conceive no disaster which calls more loudly for our sympathy and help than that of a serious injury sustained by one who is the head and main support of a family. (Cheers.) I am very glad to hear from you, Mr. Hills, as president of the hospital, that the employers of labour in the district recognise and appreciate the work which has been done by the institution, and I notice with pleasure that you, as the largest employer, have helped it very generously. (Cheers.) I am greatly impressed by finding that the hospital receives a warm support from the working classes themselves. I had learned the fact before you mentioned it in your address that thousands of them subscribe, and I have seen myself to-day, by the numbers present as delegates of their different societies, how genuine is their interest in its success. I am anxious to say to the hundreds of working men whom I see around me that my knowledge of the fact of their own labours in behalf of this hospital was one of the main causes which induced the Princess of Wales and myself to take part in these proceedings. We learn with pleasure from the president that our presence has been of service, and I thank those ladies and gentlemen whose names are mentioned in this list for their generosity. I hear with much gratification that the hospital is entirely free from debt, a fact which cannot be too highly commended, especially as such a financial position is not, I fear, altogether usual among the London hospitals. Where so many have been generous it may seem invidious to particularise individuals, but I cannot forbear from specially mentioning to you the name of Sir Donald Smith, whose generosity in his own country is well known, and who, having inquired into the work done by this hospital, has sent us the magnificent donation of £1,500. (Cheers.) I would next mention Baron de Hirsch, who, always a warm supporter of charitable institutions in this country, has forwarded a donation of £700. I think the committee and I have only paid Sir Donald Smith a well-deserved compliment by naming a ward after him to perpetuate an act of such kindness and liberality, and I am glad to find that the other two large wards have been called after Sir Donald Currie and Mr. John Coles, both of whom have been such benefactors to this hospital. I wish, in conclusion, to say how greatly I admire the unselfish devotion of the honorary staff of this institution, and these words of praise are, I am satisfied, applicable to the staffs of all our hospitals. I am informed that two gentlemen—Dr. Corner and Dr. Brownfield—who have served this hospital without fees or reward from the day of its opening forty years ago, are about to retire. They have, I am sure, earned the gratitude of all connected with the institution, and it must be a sincere pleasure to them to feel that the hospital for which they have worked so long and zealously is yearly increasing in prosperity. I heartily wish the hospital every success, and I will now ask the Princess of Wales to declare it open. (Cheers.)

The Princess of WALES then said: I have very great pleasure in declaring this hospital open.

Their Royal Highnesses then visited one of the new wards, where all those patients who were well enough had collected. The guests were received by the Matron, Miss Vacher, and the senior medical officer, Mr. Radford. The Prince of Wales inspected all the new appliances, making particular inquiries as to the precautions against fire. The other members of the Royal party conversed with the patients; one shy little sick child plucking up courage to reply to the Princess, addressed her respectfully as "Mrs. Prince of Wales," which caused some amusement.

On their return to the tent, "God Bless the Prince of Wales" was sung, and the Royal party drove off amidst loud and continuous cheers from enormous crowds who, in spite of pouring rain, lined the streets through which the procession passed.

Amongst those present were the Archbishop of Canterbury, the Lord Mayor and Lady Mayoress, Lord Knutsford, Colonel Feneran (house-governor), the Hon. Sydney Holland (chairman), Mr. A. F. Hills (president), Sir Donald Currie, M.P., and Mr. John Coles (vice-presidents), Mr. Sydney Buxton, M.P., Lady Knutsford, Lady Mary Holland, Miss Lucy Holland, Sir T. Fowell Buxton, Sir Thomas Fowler, Sir Frederick Young, Colonel Du Plat Taylor, the Mayor of West Ham, and a great many others.

### Nursing in Germany.

BY A CORRESPONDENT.

DR. N. BRÜCKNER, of Frankfort-on-Maine, has raised the cry for more nurses in his book, recently published, "Blättern für soziale Praxis." Considering the perfection of German hospital arrangements in all other respects, it is a matter for surprise that this necessity has not earlier been recognised and remedied. At present the nurses belong either to some organisation or are working on their own account. The majority belong to some religious body, and are designated Sisters in Roman Catholic countries, and Deaconesses or Sisters in Lutheran countries. There are, besides, the nurses belonging to the order of the Red Cross and to the Victoria House in Berlin. Yet, owing to the small number of these Sisters, they are terribly overworked and wretchedly paid. There is usually one Sister to each ward, with totally uneducated, inexperienced girls under her for the rough work. She begins work between five and six a.m., and seldom is off duty, except for meals, until ten p.m. This, of course, cannot be endured for any length of time, and the result is that the health of the sisters gives way, and they have to be supported by the institution to which they belong.

Dr. Brückner tells us they are paid on the same scale as washerwomen, midwives, and milliners. He complains that educated women, though having to earn their own living, never become nurses. He proposes the adoption of the English plan of three years' training, which he commends very highly. In house-to-house nursing neither the Sisters nor the Deaconesses are remunerated by their patients, the latter are expected to subscribe or give a donation according to their means to the Order to which the Sister belongs. To render the work still more unattractive, the Sisters, when entering an institution, have to contract vows, precluding them from every form of innocent amusement. The Lutheran nurses, though not bound by actual vows, are yet governed by very strict rules, and a visit to a theatre is looked upon as a gross sin. Should they marry they are regarded as black sheep for ever after.

Are these attractions for any girl who would really like to give her services? Until some alterations are made it is scarcely to be expected that many ladies will volunteer for the work. From my own experience I have known domestic servants wishing for a change take the situation of "Wärterin," that is to say attendant, in a hospital.

In Catholic countries I believe the arrangements are somewhat better. However, the Sisters can only be regarded as martyrs, living in obscurity, with no ray of sunshine save the consciousness of doing their duty and the certainty of being supported in case of sickness and old age.

Dr. Brückner's article on this subject is eloquent and to the point, and it is hoped will not be without result.

At the medical congress held lately at Breslau, it was proposed that doctors, before beginning practice, should spend a year in attendance at a hospital. This proposition will no doubt be brought into effect ere long, yet I fear it will not meet with approval on all sides. It is, however, a step in the right direction. At present the medical course is too theoretical. A little more practical experience is the very thing needed.

A Nürnberg firm is just completing an order of the Bavarian Railway Company for a number of sanitary railway carriages, fitted up with surgical instruments, bandages, a receptacle for ice, and with space for ten wounded persons in each compartment. In case of an accident these are to be at once despatched to the scene of accident. They will also be used in time of war.

### Our Examinations.

Answers to our last examination question have been very numerous, and they have come from all parts of the country. Some of them contain excellent common sense, and show that a thoughtful and intelligent grasp of the subject has been taken. Far too many competitors, however, quite ignore the general condition of the victim of an accident, devoting their entire papers to describing the external applications to the wounds. Others again advise the administration of all kinds of drugs, and recommend hot baths freely without giving any hint as to the necessity for obtaining medical sanction to both these measures when the condition of the patient is serious.

Question for May.

How would you give first aid to a person who had been badly scalded or burnt?

Prize Answer.

When a nurse is called to a person seriously scalded or burnt, she must remember the necessity for prompt measures, and in the absence of a doctor, must at once act on her own responsibility. Remembering the great danger arising from shock to the system, she should first of all sustain the patient's strength by giving some stimulant, preferably brandy, in either water or milk. If the patient is much collapsed, this nourishment should be repeated at frequent intervals. In the meantime the nurse should send for the most readily accessible dressing, and while someone is fetching carron oil (linseed oil and lime-water in equal parts), old linen, and cotton wadding, she must take every care to exclude the air from the injured surface. The patient should be laid on a couch or bed, covered with a blanket, not near the fire, and well out of draughts. If the clothes are burnt they must be very gently cut away, the utmost care being taken not to injure the cuticle by rough or hasty handling. If a large part of the surface of the body is burnt, the danger to the nervous system is increased, and the nurse must bear this in mind, and also remember not to expose the whole surface at once. The clothes having been removed, the blisters should be gently snipped, the serum absorbed by cotton wool, and the injured part then covered by old linen dipped in carron oil. Over this a layer of cotton wadding should be placed, and the dressing secured by bandages. When the whole of the injured part has been thus treated, the patient should be kept absolutely at rest; warmth must be maintained by blankets, and, if necessary, by hot bottles, and nourishment must not be forgotten. For further treatment the nurse must await the orders of the doctor for whom she has, of course, already sent. Nurse Gertrude Ward.

Question for June.

What precautions should be taken during and after an attack of measles, by the nurse in charge of the patient?

Answers, which should be written on one side of the paper only, to be sent by June 30th, to "Nursing," Editor of The Hospital, 428, Strand, accompanied by the full name and address of the writers.

### Where to Go.

Dudley Gallery Art Society's Exhibition, Egyptian Hall, Piccadilly.—The private view of the Dudley Gallery Art Society took place on Saturday. Taken as a whole, it is a very representative show of the members' work. The President, however, exhibits less conspicuously than usual, his study of wild cattle in Chillingham Park (No. 255) scarcely being up to his usual standard. The cattle themselves, which form the subject of the picture, are barely treated with sufficient dignity; they do not, indeed, stand out clearly enough from their surroundings, and their size is somewhat too diminutive, considering the general scale of the work. A very lovely picture is Mr. George Marks' "Evening Glow" (No. 266). This shows a long stretch of sunny corn-fields at harvest time, bathed in a deep warm glow. The sky is hot and cloudless. The trees stand still in the breathless atmosphere. Poppies and a luxuriance of field flowers speckle the foreground. Mr. Marks' picture is evidently the memory of a lovely and a happy day, and is by no means painted in the minor key in which much of the art and literature of 1894 are presented to the public. There are two excellent portraits in this exhibition, the work of Mrs. Marion Alexander. No. 2, "A Sherringham Fishman," is both spirited and clever. Miss Ida Kirkpatrick and Mr. David Green are the other two artists whose sketches form the most attractive portion of the exhibition. We notice a tendency to too great washiness in the studies sent in this time by Miss Evangeline Jex Blake. Miss Margaret Bernard shows decided improvement in her work, and has some pictures here which are at once strong and delightful; especially in No. 218 the artist shows herself at her best.

Afternoon Concert.—85, Lancaster Gate, on June 27th, at half-past three, for St. Joseph's Hospital for Incurable Women and Children.

The English and Continental Picture Exhibition.—June 25th.

British Home for Incurables, Crown Lane, Streatham.—H.R.H. the Princess of Wales, accompanied by H.R.H. the Prince of Wales and other members of the Royal Family, will open the new buildings on Monday, July 2nd, in the afternoon.

### Nurses for Civilians in India.

From Our Own Correspondent.

It is time, if anything is to be done in this important movement, that it should be set about, for at present, except at Lahore and Simla, no good nursing is provided for civilians in the Punjab. European civilians, and officers' wives and children, remain the only part of the community not provided for in this particular way.

The Government provides trained nurses for the army or so-called station hospitals. Civilians only in cases of severe accidents or emergency are admitted to them.

Lady Roberts has two homes, one at Kasawli and one at Murree; the former is rarely made use of. Here trained care is provided for sick officers, who are wealthy enough to avail themselves of it, but the charges are so heavy that the ordinary officer or subaltern prefers to be nursed in the station hospitals.

At present the Indian Nursing Service cannot provide nurses for all stations in India, but when their services are very earnestly needed, they are often despatched from their own stations to the less fortunate ones. A sad thing has happened at Agra; that is a station where there are no Nursing Sisters. A number of officers of the West Surrey were taken ill at the same time of enteric fever, and three died in one week. A sister was, I believe, sent from Meerut, but she was not in time to save them.

Her Excellency Lady Elgin, the new Viceroy's wife, is, I believe, deeply interested in nursing, and it is hoped that she will give a helping hand to this "up country nursing scheme," which appears to us out here to be dying a natural death.

Lady White, wife of the Commander-in-Chief, is much interested about the nursing of soldiers' wives and children.

Certainly as far as the present system works there is great room for improvement. A year ago the superintendence of these hospitals was put under the Nursing Sisters. They represent thirteen stations in India, and they do not actually nurse in the women's hospitals; so whether the superintendence means anything or not depends entirely on local administration.

The matrons of these hospitals are not always trained nurses and few are diplomaed midwives; they are also a poorly paid staff. If better pay were given, a better class of nurses could be provided, but that is not a matter for public enterprise, but rather for the Government to take up.

Measles has been very prevalent this year at Simla, and now scarlet fever has broken out. At the Simla Convent a little child has died of scarlet fever of whom it was told that coming out on the troopship to India, her mother was going downstairs with the child in her arms, her foot caught and as she felt herself falling she screamed to a soldier to catch her child; she threw it to him; it was the last word she spoke, for she herself was killed, and the poor little mite is now dead of scarlet fever.

There will be a vacancy this autumn in the Indian Nursing Service; the Deputy Superintendent of Nihow leaves in August, and there will probably be another vacancy in the spring.

☞ For The Muses' Looking Glass, Everybody's Opinion, Book World for Women and Nurses, &c., see page cix. et seq.

### The Muses' Looking Glass.

### EXPERIENCES OF PRIVATE NURSING.

I.

When a poor tired Nurse is sent out to a case,
She is hurried at once to the room
Where her patient is lying with a woe-begone face,
For he thinks he will die very soon.

II.

"Oh! Nurse, there you are, we are glad you have come,"
The thankful relations exclaim,
"For we hear that trained nurses are wonderful folk
And have made for themselves a great name."

III.

"They can live without food—the story so goes,
They never require fresh air;
They can do without sleep for six weeks at a time,
Not even to doze do they care.

IV.

"*You*, no doubt, Nurse, are one of these wonderful folk,
We hope you will now act as such;
Of course we'll allow you one meal in the night,
As we're poor we can't spare very much."

V.

They deliver these timely and obvious remarks,
Then these worthy relations depart
To take their nice tea—eggs and hot buttered toast,
Poor Nurse longs for some from her heart!

VI.

She looks at her patient, he is now sleeping well,
With a calmer expression of face;
No wonder, they've just made him swallow a draught
Which *might* make him a very bad case!

VII.

Fifteen minims were ordered, they increased it to 30,
They mean well, and do all for the best;
They think it is better to give him enough,
And procure a good sleep and night's rest.

VIII.

They have finished their tea—Nurse hears them come up,
She thinks "I shall now have no peace."
His mother, his wife, his sisters, his aunts,
His mother-in-law and his niece!

IX.

She was right, for they all began cackling like hens,

The mother-in-law worst of all:

"I've had great experience in nursing," she says,

"If you want help, Nurse, 'tis *me* you're to call.

X.

"Have you taken his temperature yet, and his pulse,
Have you tried if he'll drink any more?
His pulse was 280 last night!
His temperature 204° !!

XI.

"I hope you will see that his feet are quite warm;

Will you give him the medicine now due?

Don't forget 'respiration' when taking the pulse,

Last night 'twas 102!

XII.

"You're to give him some milk and some stimulant too,

And the medicine comes due every hour;

The poultice is due at eleven o'clock;

Don't give him the milk if it's sour.

XIII.

"The powders are given twice during the night;

The pills are at twelve, four, and eight;

He's to get some beef-tea and a beaten-up egg.

Now, I'll go to my bed as 'tis late."

XIV.

Nurse hopes the directions are over at last,
If they'd *all* go to bed 'twould be nice,
But the others begin them all over again
Each with her own bit of advice.

XV.

Nurse now thinks it time to give them a hint,
Of her thoughts she does not make secretion;
"If my patient must swallow all these in a night
He will certainly die of repletion!

XVI.

"Just allow me to use my own judgment with him,
And I think you will find he'll do well;
I am sure you are all very tired to-night
By your looks I can easily tell."

XVII.

Oh! what a wonderful stroke of good fortune,
They take this broad hint in good part,
And Nurse in the pleasure of seeing them go,
Says "Good-night" with a jubilant heart.

XVIII.

She now takes a look at *her* stores for the night,
The bread would just half feed a lark;
An egg—she's afraid it is not very good;
It might have been laid in the Ark.

XIX.

Just one quarter ounce of very salt butter,
An invisible atom of tea;
Two small lumps of sugar—two ounces of milk,
A sausage, the size of a pea!

XX.

"Such is life" thinks poor Nurse, "*out*-nurses, indeed,
Would need to be wonderful folk;
For to live without food, without air, without sleep,
Is beyond my idea of a joke."

XXI.

"Oh! If only I could be a 'Pro' once again,
And escape being sent to a case;
I'd work like a 'Pro,' and not ask to be 'Staff,'
And I'd like to remain in one place."

XXII.

"That place is the —— Hospital,
You doubtless have heard of its fame;
It is there I would stay—let the others go out,
And make for themselves a great name."

—*A Victim.*

### Notes and Queries.

### Queries.

(81) *Stewardess.*—Kindly tell me how to apply for post as stewardess.—*Margate.*

(82) *Snoring.*—I should be glad to know of a remedy for snoring.—*A Nurse.*

(83) *Address.*—Will you kindly give me the address of the Workhouse Nursing Association, I cannot find it anywhere.—*B. K. D.*

(84) *Male Nurse.*—Kindly tell me where I can get trained as a male nurse.—*C. Morris.*

### Answers.

(81) *Stewardess (Margate).*—Write to any shipping company, the addresses are in daily papers, and ask for form of application to fill up.

(82) *Snoring (A Nurse).*—Consult a doctor as to the cause of the snoring.

(83) *Address (B. K. D.).*—Address Hon. Sec., Workhouse Infirmary Nursing Association, 6, Adam Street, Adelphi, Strand. "Burdett's Hospital Annual" is the best book of reference for institutions of all kinds.

(84) *Male Nurse (C. Morris).*—We regret to tell you that no General Hospitals in England train male nurses.

### Wants and Workers.

Can any one give or lend an old (sound) water bed for use of a bed-ridden old man?—*Parish Nurse, Tarporley, Cheshire.*

### Everybody's Opinion.

### NURSING IN SOUTH AFRICA.

"Amafutle" writes: I see in your issue of April 7th that "Rabbi" wishes for reliable information on the prospects of a nurse starting on her own account at Durban, Johannesberg, Cape Town, or Port Elizabeth. I, therefore, write, in case no better information has been offered. Though my work has mostly been in Maritzburg, I came out to an institution for nurses towards the end of 1893. I did not like the institution and only remained at it for a year, during that time I had only work for seven months out of the twelve. When I left I at first got very little work owing to not being able to advertise, as I had come out under a three years' agreement, but the committee of the institute kindly gave me permission to work and then I advertised. I have since then (November, 1893), had plenty of work, and since Christmas have been constantly employed. However this is the sick season—from now till September or October it is probable there will be very little work for nurses except confinement cases. I have nursed in Maritzburg, Durban, and up country on farms. I do not feel sure that trained nurses are appreciated. Some of the doctors here do not like them, and many people prefer some woman to cook and look after things in the house, as few people here have white servants, and the Kafir boys who are the servants as a rule, do not work well except under supervision and for their own "Boss" I know two doctors here are of opinion that a trained nurse could not find enough work to keep her in Maritzburg. I think if a nurse could afford to wait she might at last make a living in Durban. I find living (board and lodging) come to about six pounds a month; when at work I get two guineas a week, washing, and travelling expenses. I hear that Johannesburg is overrun with nurses, of a sort, but very few are hospital trained. I also hear that at the Cape they are always ready to take private nurses on to the staff at the hospital, so I presume there is a demand for them. I believe a nurse with introductions to doctors might get plenty of work. The houses are mostly small and very inconvenient, it is difficult to keep the patient quiet, as nearly all the rooms open on to a verandah or into other rooms. Kafir servants do not like taking orders from a stranger, and the nurse just out from home cannot make the Kafirs understand what she wants. I find the people most kind and hospitable, they treat the nurse as one of the family, and whenever I have nursed up country I have invariably been kindly invited, when leaving, to return and stay as a guest, and I have already had a very pleasant holiday with friends on a stock farm. I know nothing, by hearing or otherwise, about Port Elizabeth.

### DISTRICT NURSES.

Miss Mary Mason writes: I have read with much interest the discussion upon the amount of training and the qualifications necessary for a district nurse. I am a trained hospital nurse of twelve years' standing, and have had charge of medical and surgical wards in four large hospitals, and have also worked in the district. I am pleased to find that there is a movement on foot for providing the sort of nurses who are really most important to the poor—a strong, able-bodied young woman with a good head upon her shoulders, who can make poultices, dress wounds, and who is not above clearing up, and doing a great deal of hard work besides the actual necessities of the patient. A great deal is said about the skill and qualifications necessary for nursing the poor, and in a measure this is true, but three months' careful training given to the right sort of woman is ample. A district nurse always works under a doctor—therefore the responsibility of taking the lead is not hers. In London especially all the most important cases go at once to the hospital. The ordinary run of district work is composed of chronic cases, paralysis, ulcerated legs, senility, phthisis, &c., varied by a very occasional typhoid case (which for obvious reasons would be better in the hospital), and now and then an operation, which also would be better away from the slums, and always under the supervision of nurse and doctor. These very highly-trained nurses who are overstocking our districts require high salaries, and where we could have two young, strong, willing nurses we now often have to pay one nurse, no longer young, and therefore quite unfitted for hard work and perpetual physical exercise, a salary of £50 or so and everything found to do what could be better done by others. A good ovarian nurse, for instance, is absolutely necessary for her own kind of work, but when placed in the district she can make a bed and wash a patient well and carefully, but so can a bright, capable young woman of less experience, who would ask half the salary required by the former, and many poor parishes now unable to have a nurse would gladly accept the services of a nurse within their means, whereas now they must obtain the very precarious nursing provided by large societies, who send a nurse or not as they choose, and who, if the case is not what they consider "good," simply give it up. It is absurd to say that because a rich man must have the highest skill in nursing so also must a poor man. A rich man always has a good dinner, so should a poor man, but he lives and thrives on what he is accustomed to, and probably a good dinner of the sort enjoyed by the rich man would not agree with him at all. A poor man's wife is not an educated woman, though the wife of the rich man is, but she is more suited to his needs; and I maintain that a loving, capable woman, with three months' thorough hospital training in simple minor details of nursing, and her heart in her work, is of as much use amongst God's poor as the finest lady in the land with twenty years' experience.

*** This writer's experience of district work appears to be somewhat limited, otherwise she would not venture to assert that all the most important cases go at once to hospital. The reports of all associations which provide *trained* nurses show a very different state of things. A just estimate of the difference between skilled and unskilled nursing can hardly be formed by one who thinks three months in hospital adequate training. We are content ourselves to be guided by that great majority of experienced workers who endorse the Queen's view of district nursing, viz., that as the lives of the poor are as valuable as those of the rich, they should be supplied with equally skilled attention in sickness.

### Royal Asylum, Morningside, Edinburgh.

The following passed the examination for the nursing certificate of the Medico-Psychological Association: Nellie Clark, Maggie King, Willimina Milne, Isabella Silver, Mary Ann Watson; Alexander Crockott, Thomas Douglas, John Hughson, Andrew Macdonald, John Macdonald, Thomas Macdonald, and John C. Smith.

### Minor Appointments.

Wembley Hall, Middlesex.—Miss M. L. Lockhart has been appointed to the District of St. John's. She has won the confidence of doctors and patients by her good nursing during past winters at the Home for Invalid Ladies at San Remo. We wish her success in her new sphere of usefulness.

Keighley and Bingley Joint Hospital Board.—Mr. and Mrs. Overshott (trained nurse) have been appointed Porter and Matron at this hospital. They have held previous appointments at Ecclesall Bierlow Temporary Infectious Workhouse Hospital.

### Presentations.

The Nurses of the James Murray's Royal Asylum, Perth, recently presented their Matron, Miss Mountford, with a silver inkstand on her birthday.

### The Book World for Women and Nurses.

[We invite Correspondence, Criticism, Enquiries, and Notes on Books likely to interest Women and Nurses. Address, Editor, The Hospital (Nurses' Book World), 428, Strand, W.C.]

A Study in Colour. By Alice Spinner. (London: Fisher Unwin. 1894. Pseudonym Library.)

Quite unique in point of its matter is Miss Spinner's little story. We feel through its perusal to be brought in closer sympathy with a race from which we have ever been divided, through the great gulf fixed between the white man and the black, a line of demarcation which Miss Spinner shows us is due to colour alone, but realised by the negro in an exaggerated degree. This last addition to the Pseudonym Library brings the fact home to us in an insistent manner. It is written and told by one who knows the negro as he is, with all his virtues and all his faults. In reading the simple little story of a far-away West India isle, the authoress puts us, as its were, so in touch with her immediate surroundings, that our own are for the time being forgotten. Very pathetic is Justina's history, the dusky servant girl, whose faithful labours reflect in no very creditable manner on the service of a more civilised race. We realise anew through these pages the negro's attitude towards the white man, how immeasurably high they set the value of the white skin, not alone on earth, but in eternity. The black Justina's words convey the general spirit in this respect. Her mistress's only child, the young master, was just dead. He was the object of her adoring slavery, and in her efforts to comfort the mother, the dusky servant explains, "You see de blessed little massa 'gain in heaven; but what is dere for me? I know dey say we go up dere wid you just de same, but do you really tink that, missus? I know we all God's children, so dey tell us in de church, but our ways are not your ways, nor our thoughts your thoughts. . . . I love you, my tredsor, for ebber. Chance I see you far off one day, but is it likely in de blessed heaven you need your poor old nurse?"

A Modern Amazon. A Novel by George Pacton. (London: Osgood, McIlrain and Co.)

This is a novel in which a single idea is ridden to death. The idea may be a good one, but it is hardly worthy of being spun out into two volumes. The tale tells how a somewhat eccentric, philanthropic, but otherwise an uninteresting doctor falls in love with Regina, the heroine. Regina is a young lady with ideas, she was born to wealth, but when the story opens she is earning her living as secretary or assistant to the editor of a society paper. The editor of course falls in love with his assistant, a love which is unreciprocated by the passionless young Amazon; and in spite of this declaration of love, the young lady, in what may be considered a somewhat undignified manner, retains her post. Later she meets with Dr. Kenyon, who apparently sees few charms in her, until a mutual friend suggests that he ought to marry a young lady of such surpassing virtues. He accordingly proposes, and Regina, who has a considerable respect for his abilities, accept him, on the following cold-blooded terms: They are to remain friends and companions, respect but not love is to form the bond of their union. The doctor, after some consideration, accepts the terms, since in his blind philosophy he believes that terms which controvert the laws of nature could never condition the conduct of man and wife. The tale continues to prove that Regina knew what she was about, and that the doctor was altogether out in his calculations. The latter, who is an honourable gentleman, tries every legitimate suggestion made by his friends to alter the conditions under which he is doomed to conjugal celibacy, but the monotony of their life is only relieved by occasional squabbles, which ends in a temporary separation; and the tale ends as the quarrel is made up, and the platonic absurdities vanish into thin air as Kenyon clasps his wife to his bosom.

There is much that is true, as there is much that is false to nature in the novel. Regina's want of dignity, as well as her ignorance of the world, is hardly compatible with the high intellect and worldly experience with which she is credited.

The Little Man Island. By Hall Caine. (Printed by Richard Johnson, Limited, Manchester. 1894.)

Now that the holiday season is fast approaching, one is glad of suggestive hints what to do, and where to go. A small volume has just been published which offers an advantage of this kind. It is entitled "The Little Man Island," by Mr. Hall Caine, who deals entirely with the opportunities offered to those visiting the Isle of Man. Certainly, small as it is, the Home of the Manx seems to possess much that is of interest to those who visit its shores. Then it is quite easy of access, and the steam service is frequent and good. It has been said, and Mr. Hall Caine reiterates the statement, that there is nothing more beautiful than the Isle of Man when it is approached from the English side and seen in the late afternoon. Before one's eye stretch the line of mountains, and more immediately in the foreground the lights of Douglas Town. To all who contemplate a pleasant little summer trip we confidently recommend a perusal of the above book.

### MAGAZINES FOR THE MONTH.

The Cornhill Magazine for June gives us the conclusion of "With Edged Tools," a conclusion which, though to be anticipated, is not, strictly speaking, quite a happy one. The story is both clever and attractive, and our interest has been well sustained in it throughout its perusal. A paper on "The German Military Examination" is contained also in this number. Had the various items herein recorded been contrasted side by side with other international army requirements, it would have proved itself both a significant and an interesting comparison.

The Humanitarian continues to sustain the reputation it has deservedly gained of an organ that does not fear facing the problems of the day. Those difficult social problems which it is always easier to dismiss from our minds as harrassing and unpleasant subjects. Without being called attention to, a betterment cannot be expected of many existing matters, and the Humanitarian does not shrink from the task which it has set itself. Professor Victor Horsley, F.R.S., deals in this issue with the "Vivisection Controversy," in reply to Bishop Barry's antagonistic article in the April issue; and labour and social questions are treated by Sir John Gorst in the instance of "An Interview with the Statesman."

Cassell's Family Magazine has a capital chapter on "Musical Gestures," by Professor Bridge, Mus.D., where he explains an absolutely simple method of teaching beginners the elements of music. The whole course of this instruction is contained in Dr. Bridge's "Musical Gestures," published by Novello and Co.

The Fireside Pictorial Magazine, in spite of its name, is by no means a periodical which confines itself to articles which are suitable only for reading around the fire. The June number contains much that is worthy a longer mention than can be afforded by our limited space here. A new serial story commences in this issue, written by Miss Giberne, and entitled "Life Tangles."

